# Red-Ox Energy Partitioning of Light-Driven Electrons: From Laser Ablation to Plasmonics

**DOI:** 10.3390/mi17080988

**Published:** 2026-08-21

**Authors:** Haoran Mu, Hsin-Hui Huang, Tomas Katkus, Nguyen Hoai An Le, Jurga Juodkazytė, Yoshiaki Nishijima, Saulius Juodkazis

**Affiliations:** 1 Optical Sciences Centre, Swinburne University of Technology, Hawthorn, VIC 3122, Australia; hsinhuihuang@swin.edu.au (H.-H.H.); tkatkus@swin.edu.au (T.K.); ale@swin.edu.au (N.H.A.L.); 2 ARC Training Centre in Surface Engineering for Advanced Materials (SEAM), Swinburne University of Technology, Hawthorn, VIC 3122, Australia; 3 Center for Physical Sciences and Technology (FTMC), Saulėtekio Ave. 3, LT-10257 Vilnius, Lithuania; jurga.juodkazyte@ftmc.lt; 4 Institute of Advanced Sciences, Yokohama National University, 79-5 Tokiwadai, Hodogaya-ku, Yokohama 240-8501, Kanagawa, Japan; nishijima@ynu.ac.jp; 5 PRESTO (Precursory Research for Embryonic Science and Technology), JST and Institute for Multidisciplinary Sciences, Yokohama National University, 79-5 Tokiwadai, Hodogaya-ku, Yokohama 240-8501, Kanagawa, Japan; 6 Laser Research Center, Physics Faculty, Vilnius University, Saulėtekio Ave. 10, LT-10223 Vilnius, Lithuania

**Keywords:** laser reduction of titania, water splitting, femtosecond laser ablation, hot electron injection, reactive oxygen species

## Abstract

In femtosecond-laser processing of titania in water, light can induce reduction and oxidation simultaneously. We follow this redox energy partitioning, in this perspective, from colloidal titania synthesis to hot-electron devices. Femtosecond ablation/fragmentation of an aqueous anatase suspension (515 nm, 230 fs, 5μJ, fluence F≈25.5 J cm^−2^/pulse at clamped intensity ∼$10^{13}$ W cm^−2^) yields surface-reduced, Ti^3+^-rich bluish TiO_2−x_, while the same optical breakdown generates reactive oxygen species (ROS), among them H_2_O_2_ and HO^•^ radicals, which compete by re-oxidising Ti^3+^. When the reduced titania is decorated with plasmonic nanoparticles (e.g., Au), an n-type plasmonic photo-electrode is realised: sp hot electrons are injected over the Schottky barrier, while the deep *d*-band supplies oxidising holes. The oxygen evolution reaction (OER) proceeds in stages at potentials well above the formal 1.23 V via the two-electron peroxide route (∼1.77 V) or, for sufficiently energetic holes, via the one-electron HO^•^ route (∼2.7 V). In a biased cell, H_2_ evolves on Pt through the adsorbed (H_2_^+^)_ad_ intermediate. The same Au/semiconductor physics on silicon enables sub-band-gap hot-electron photo-detection. Energy-level diagrams (flat-band and in-contact) and the sp- vs. *d*-band origin of the injected carriers are discussed.

## 1. Motivation: Solar Fuel

Photocatalytic and photoelectrochemical water splitting store sunlight in the H-H bond, but the efficiency of any such device is set by the fate of the electrons and holes created on light absorption. Time-resolved spectroscopy was used to benchmark oxides and to show that performance is governed by three coupled processes on very different timescales: (i) ultrafast band-edge and bulk recombination that destroys e-h carriers, (ii) the slower spatial *separation* of electrons and holes, often driven by the space-charge field of a junction or by an applied bias, and (iii) trapping at surface states where holes in contact with water either drive oxidation or are lost [1,2,3,4]. A recurring lesson is that the oxygen evolution reaction (OER) needs *long-lived* holes for slow O_2_-evolving chemistry and that buying lifetime costs voltage [5]. The intrinsic lifetime is in turn tied to the metal centre, where a sub-picosecond relaxation through metal-centred ligand-field states quenches carriers and explains why high-dielectric $d^{0}$-materials such as SrTiO_3_ hold charges far longer than hematite and perform so well [6,7,8]. It was shown what those design rules yield in practice having extended light harvesting into the visible with (oxy)nitrides and oxysulfides [9,10,11,12,13] and pushed SrTiO_3_ to near-unity quantum efficiency by suppressing recombination [14]. Powder photocatalysts were taken out of the laboratory and immobilised as fixed photocatalyst sheets, reaching above 1% solar-to-hydrogen conversion [15]. The 1 m^2^ panel under natural sunlight [16] and a 100 m^2^ outdoor panel array run safely for months with membrane gas recovery [17,18]. Solar water splitting on metal oxides and nitrides directly advances UN Sustainable Development Goal 7 (Affordable and Clean Energy) by offering a route to renewable green hydrogen, while also supporting SDG 13 (Climate Action) through carbon-neutral fuel production [19].

## 2. Light as a Trigger for Reduction and Oxidation

Since the discovery of photoelectrochemical water splitting on TiO_2_ [20], titania has been the archetypal photocatalyst, and a major route to extending its activity into the visible has been *reduction*: introducing oxygen vacancies and Ti^3+^ (Ti(III)) centres to make “black” or colored TiO_2−x_ [21,22]. The central theme of this perspective paper is that, in the processes used to make and to use such materials, light simultaneously induces reduction and oxidation: the absorbed photon energy is partitioned into reducing and oxidising species or carriers. This redox energy partitioning recurs from colloid synthesis to hot-carrier devices.

Pulsed-laser processing of materials in liquids is a versatile, surfactant-free route to colloidal metal-oxide nanomaterials [23,24]. In pulsed laser ablation in liquid (PLAL), a solid Ti or TiO_2_ target is ablated. In laser melting/fragmentation in liquid (LML/LFL), an existing suspension is irradiated, causing each particle to be transiently melted and re-solidified. In both, the reactive aqueous environment and the non-equilibrium quench imprint oxygen vacancies and Ti^3+^, producing reduced TiO_2−x_ with a blue-to-black coloration [22,25,26,27]. The pulse duration sets the physics: nanosecond pulses act thermally with a large heat-affected zone, whereas femtosecond pulses drive non-linear (multiphoton/avalanche) absorption and optical breakdown before heat diffuses, favoring small, defect-rich particles and reduction of commercial anatase without chemical reductants [25,26]. The color tracks the degree of reduction [22].

These femtosecond pulses also fragment colloids. It was shown that fs-ablation of gold in water gives bare colloids in two regimes: (i) small (3–10 nm), nearly monodisperse colloids at low fluence and (ii) large particles with a broad size distribution, formed via plasma heating at high fluence [28]. Size is then controlled with the white-light supercontinuum that an intense fs-pulse self-generates in the liquid: spread along a self-focused filament and overlapping the colloid plasmon, it fragments particles in a 3D volume so that they re-coalesce smaller and more stable, tunable by fluence [29]. This method was used to make ultra-pure water-dispersed gold [30], and oxidative fragmentation pushes gold below 3 nm [31]. Comparable down-sizing is reachable by ns-laser fragmentation [32] or by non-laser routes (cavitation, discharge, milling).

The oxidising face of light is equally important. Optical breakdown of water is radiolytic: it produces reducing species (short-lived $e_{aq}^{-}$, H^•^-radical (an atom) as well as H_2_) but also strong oxidisers (HO^•^, H_2_O_2_), so the very pulse that reduces titania can also re-oxidise it. The same energy partitioning appears in plasmonics: decay of a localized surface plasmon yields hot electrons (from the gold sp band) that can reduce, and hot holes from the deep *d*-band that can oxidise [33,34,35,36].

Electron–hole generation and charge separation are, of course, the textbook basis of semiconductor photocatalysis. The perspective taken here is narrower and more specific: we follow how the absorbed photon energy is *partitioned* between sp-band electrons acting as reductants and deep *d*-band holes acting as oxidants, and we show that this same partition provides a single organising principle across three otherwise disparate settings: femtosecond colloid synthesis, plasmonic photoanodes, and hot-electron detectors. The aim of this perspective is to trace this redox energy partitioning through these settings and to make explicit the energetics that decide, in each, what the device can do.

Building on these ideas, the remainder of the paper follows one thread through three settings: (i) femtosecond laser reduction of anatase and the plasmonic enhancement of its activity; (ii) reduced titania decorated with gold as a photoanode for water splitting; and (iii) the same Au/semiconductor plasmonics enabling sub-band-gap (sub-wavelength) detection on silicon.

## 3. Results and Discussion from the Electron Transport View

The three settings discussed below are connected by a single physical thread: in each case, the absorbed photon energy is partitioned between sp-band electrons, which act as reductants, and deep *d*-band holes, which act as oxidants. In the femtosecond laser synthesis of reduced titania (Section 3.1), this partition occurs within the laser-driven plasma itself. When the same material is assembled into a plasmonic photoanode (Section 3.3), the two carriers are directed to separate half-reactions: sp electrons reduce protons at the cathode while *d*-band holes oxidise water at the anode. Transplanting the identical Au/semiconductor interface onto silicon (Section 3.4) moves the useful output from a chemical fuel to an electrical photocurrent, but the underlying sp/*d* partition remains the same.

### 3.1. Laser Reduction of Anatase and Plasmonic Enhancement of Its Activity

**Figure 1:**
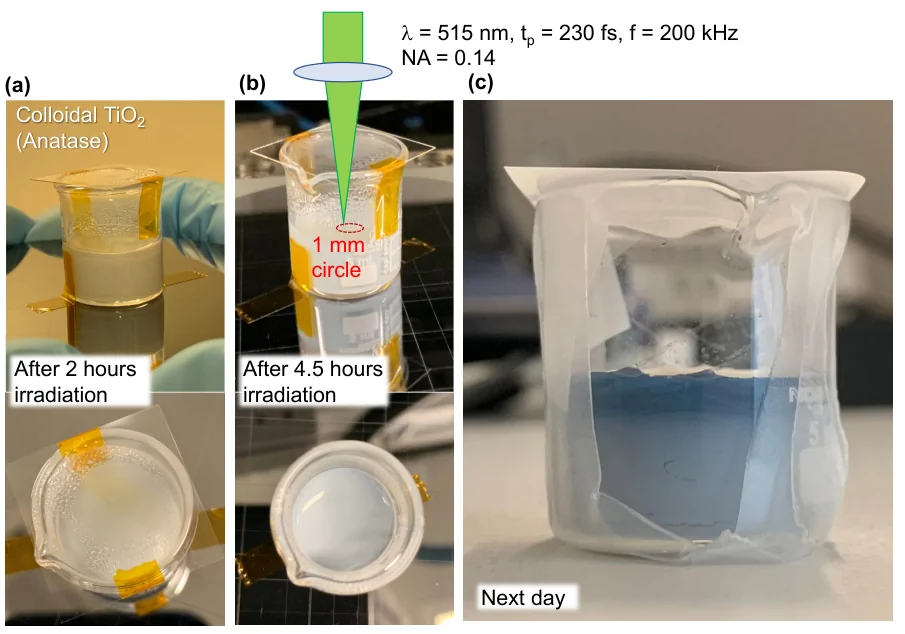
Femtosecond-laser processing of the titania suspension. (**a**) Suspension after 2 h of irradiation; (**b**) after 4.5 h, showing the progressive blue coloration; (**c**) “next day”: after standing for ∼24 h, the material separates under gravity, the darker (more strongly reduced) fraction remaining suspended while lighter material settles into colour-graded layers. The beam was held stationary at the liquid surface (no raster scan); the ∼1 mm circle marks the path along which the beaker was moved to agitate the suspension; the beam was interrupted every 2 min and the beaker swirled 30 times. Full processing parameters are given in Section 3.1 and on the figure. Two irradiation sessions were used: 2 h (60×2 min) then 2.5 h (75 iterations), with a ∼0.5 h gap.

**Synthesis.** Following [37], an aqueous suspension of commercial anatase microparticles (∼40μm) at 15 mg mL^−1^ was irradiated at the liquid surface with a 515 nm, 230 fs laser (Pharos, Light Conversion) at 200 kHz, using 5μJ pulses focused to a ∼5μm spot, with periodic agitation; the suspension darkened progressively with irradiation time (Figure 1a,b) and, on standing overnight, separated under gravity into colour-graded layers (Figure 1c).

**Fluence, intensity and clamping.** For a focal disk of diameter d=5μm the area is $A=\pi {\left(d/2\right)}^{2}=1.96\times 10^{-7}$ cm^2^, so $E_{p}=5\;\mu$J gives a fluence $F=E_{p}/A\approx 25.5$ J cm^−2^ and, at τ=230 fs, a geometric average intensity $I=F/\tau \approx 1.1\times 10^{14}$ W cm^−2^ (111 TW cm^−2^; for a Gaussian pulse the peak intensity is twice larger); the average power is 1.0 W and the photon energy 2.41 eV. This is deep in the optical-breakdown regime (water breakdown $∼10^{13}$ W cm^−2^, titania ablation ∼1 J cm^−2^). However, the peak power (∼22 MW) far exceeds the critical power for self-focusing in water ($P_{cr}=3.77\lambda^{2}/8\pi n_{0}n_{2}\approx 1$–4 MW), so the beam filaments and Kerr self-focusing are balanced by plasma defocusing, which clamps the in-filament intensity to the order of $10^{13}$ W cm^−2^, largely independent of pulse energy [38,39]. The same self-focusing also generates the white-light supercontinuum. The geometric value is thus an upper bound, with the dose spread along the filament. The superheated, oxygen-deficient plasma and reaction with water (Ti−OH, Ti−H) create the lattice disorder, oxygen vacancies and Ti^3+^ of the bluish product [26,27,37].

**Competing oxidative chemistry (ROS).** Radiolysis in the fs-laser filament in water also makes oxidisers (reactive oxygen species, ROS): HO^•^ radicals and, by 2 HO^•^ → H_2_O_2_, hydrogen peroxide, ($E^{\circ }\approx +1.78$ V), which re-oxidise Ti^3+^ to Ti^4+^ (here Ti^3+^ denotes Ti(III) in the reduced surface oxide rather than an ion in the solution phase, i.e., Ti_2_O_3_ + H_2_O_2_ → 2 TiO_2_ + H_2_O). The couple that actually operates here is not the dissolved TiO^2+^/Ti^3+^ pair, which has no meaningful existence in near-neutral water because Ti(IV) hydrolyses and precipitates above pH ≈1, but the solid-oxide Ti(IV)/Ti(III) couple of the reduced surface layer, 2 TiO_2_ + 2 H^+^ + 2 e^−^ → Ti_2_O_3_ + H_2_O. For the hydrated oxides, its tabulated standard potential is $E^{\circ }=-0.091$ V vs. the standard hydrogen electrode (SHE) at pH 0 (Table 1) [40]. Re-oxidation of this couple by H_2_O_2_ (+1.36 V) and HO^•^ (+2.32 V vs. SHE at pH 7), and even by dissolved O_2_ (+0.82 V), is therefore strongly downhill, which is why the competition caps the reduction at blue and accounts for the ageing of the colloid in aerated water. Because the Ti(III)-Ti(IV) redox couple resides at the surface, reduction is fast and local in the hot bubble, while surface-bound H_2_O_2_ oxidises. Oxidation and reduction proceed on the same surface, so H_2_O_2_ is formed at the interface; however, it could be transported to the surface from the solution. This plausibly explains why the product plateaus at blue rather than black and why reduced titania ages in aerated water, where TiO_2−x_ can also be oxidised to TiO_2_ by dissolved oxygen. Peroxide is useful for oxidative fragmentation [31], and the oxidising channel can be suppressed (inert atmosphere, HO^•^/H_2_O_2_ scavengers, lower fluence) to favour deeper reduction. The energetics of the radical channel sets the scale of the oxidising power available: one-electron oxidation of water to the HO^•^ radical requires potentials as high as +2.7 V [41], which makes HO^•^ the strongest oxidiser generated in the filament. This is the synthesis-stage face of the light-as-oxidiser theme. The oxidising species themselves (HO^•^, H_2_O_2_) were inferred rather than directly assayed here: being transient or diffusible, they lie outside the scope of the measurements in Ref. [37]. Their action is supported indirectly by the saturation of the reduction at blue, the persistence of the darkest fraction in suspension, and the ageing of the reduced colloid in aerated water. Direct assays (Ti(IV)-peroxo or Amplex Red for H_2_O_2_, coumarin or terephthalate fluorescence trapping for HO^•^, and DMPO spin-trapping EPR for both) as a function of fluence and atmosphere, correlated against the Ti^3+^/Ti^4+^ ratio, would close the point.

**Figure 2:**
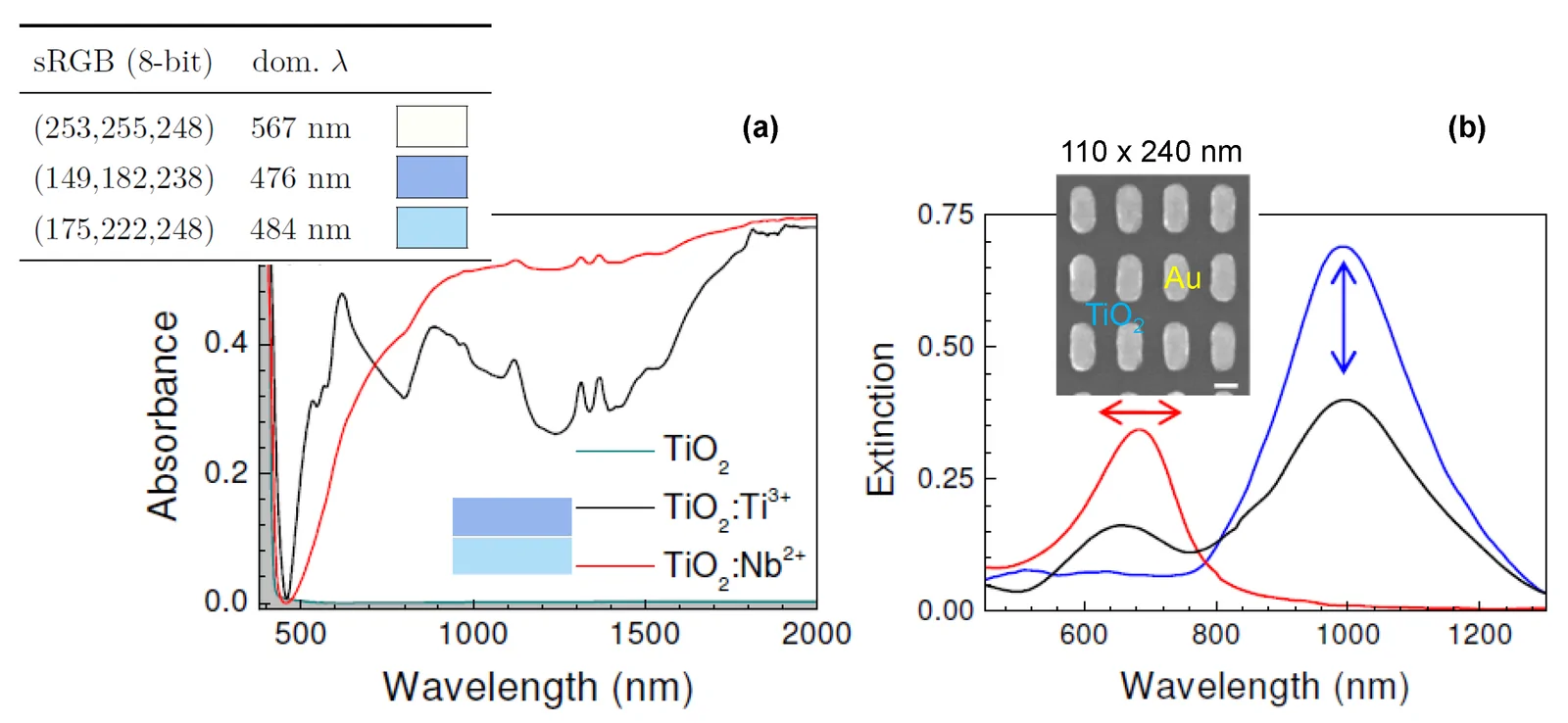
Plasmonic solar cell. (**a**) Absorption spectrum of different titania substrates: n-type Nb-doped TiO_2_ (Furuuchi Chemical Co., Tokyo, Japan), reduced TiO_2_:Ti^3+^ (Shinkosha Ltd., Yokohama, Japan), and undoped TiO_2_; thickness of samples was 0.5 mm. The top-inset shows color equivalence (sample’s appearance) and RGB coefficients of the chromaticity diagram. (**b**) The extinction spectrum of an Au nano-rod pattern fabricated on the untreated TiO_2_ single crystal measured under unpolarised and polarised illumination in water; polarisation is marked by arrows. This panel shows the spectral overlap and polarisation selectivity of the plasmon resonances, which are a precondition for carrier injection rather than a measurement of enhanced photoactivity. The inset shows an SEM image of the pattern of gold nanorods of a 240×110 nm^2^ footprint (Replotted from Ref. [43]).

**Characterisation.** The product separated by density/color into light-blue, dark-blue (laser-ablated “LA-mid”) and dark-blue/black (“LA-top”) fractions (Figure 1), the darkest staying suspended longest [37]. X-ray photoelectron spectroscopy showed the signatures of reduction in every fraction: the O 1s lattice-oxygen peak shifted up by 0.2–0.3 eV with a new ∼532 eV oxygen-vacancy component, and the Ti 2p doublet broadened and shifted to lower binding energy, indicating Ti^3+^ alongside Ti^4+^. The Ti^4+^ component is the oxidised species; what distinguishes the fractions is the Ti^3+^/Ti^4+^ ratio rather than the presence of either, and this ratio is the quantitative signature of the capped reduction, highest in the darker LA-top fraction. X-ray absorption at the Ti K-edge (XANES/EXAFS) was, by contrast, indistinguishable from anatase, so the reduction is surface-confined while the bulk remains crystalline anatase, as expected from a commercial-anatase feedstock. The visible-to-IR absorption responsible for the blue color (Figure 2a) is the optical fingerprint of the Ti^3+^/vacancy states, and a CIE-1931 evaluation of it returns a blue transmitted chromaticity (similar color as in Figure 1). Under visible light, the colloid degraded ∼79% of methylene blue in 3.5 h (pseudo-first-order $k_{\mathrm{app}}=7.1\times 10^{-3}$ min^−1^) with enhanced dark adsorption relative to the parent anatase; the low yield of the darkest fraction (∼5–6 mg) reflects losses to the container walls and to incompletely fragmented particles that sediment rapidly, and was insufficient for morphological (SEM/TEM/DLS) or N_2_-adsorption (BET) characterisation; the bulk crystalline structure was instead confirmed by XANES/EXAFS at the Ti K-edge (indistinguishable from anatase), consistent with a surface-confined reduction. The photocatalytic tests were performed with a 100 mg L^−1^ suspension in 40 mL of methylene blue solution (5 mg L^−1^) under a Philips 19 W cool-daylight LED placed ∼15 cm from the sample. Full experimental details are given in [37].

**Plasmonic coupling to the reduced oxide.** Reduction of titania broadens its absorption, but the trapped carriers recombine readily. Decorating the reduced oxide with gold nanoparticles adds a route to harvest visible light and to separate charge. Gold nanorods on titania show transverse/longitudinal plasmon extinction bands (∼680 and ∼1000 nm, Figure 2b) that can be tuned to overlap the substrate absorption. Figure 2b establishes this spectral overlap and the polarisation dependence of the resonances; it is a precondition for injection, not a measurement of enhanced photoactivity. The evidence that plasmon-induced charge transfer rather than heating drives the chemistry is the action spectrum, in which the photocatalytic or photocurrent response tracks the localised-plasmon absorption band; this is established for Au/TiO_2_ in Refs. [33,42] and measured for this electrode geometry by Nishijima et al. [43,44]. Plasmon decay then injects carriers across the metal-oxide interface; the mechanism of plasmon-induced charge separation was first demonstrated for Au/TiO_2_ [33] and is now central to plasmonic photocatalysis [34,35,36].

### 3.2. Selective Surface Activation Induced by Laser

Selective Surface Activation Induced by Laser (SSAIL) forms conductive copper (other metals) traces on undoped dielectrics: polymers, glass, ceramics and silicon in three steps: ultrashort-pulse laser modification of the surface, chemical activation in a catalytic precursor (typically AgNO_3_) solution, and autocatalytic electroless plating [45,46]. Its central, still-unsettled step is that the laser-modified regions spontaneously reduce Ag^+^ to metallic Ag^0^ seeds without any external reducing agent, while adjacent untreated areas remain inert. Existing accounts attribute this to laser-generated “electron-donating centres” non-bridging oxygen, oxygen vacancies and silanol ≡Si–OH groups on glass, and radical or carbonaceous species on polymers, yet the effect resists topographic explanation, since active and inactive surfaces can appear morphologically identical [45,47]. A redox reading, discussed here, offers a unifying picture: femtosecond breakdown partitions the absorbed photon energy into reducing and oxidising channels simultaneously, so the pulse writes not a chemical or topographic change but a map of local surface redox potential, as in the case of trapped Ti^3+^-like mid-gap donor states whose formation is capped by competing reactive-oxygen-species back-oxidation. In this view, the activation bath simply titrates the written potential: Ag^+^/Ag^0^ (+0.80 V vs. SHE) is reduced only where the laser has driven the local potential below its level. The framework rationalises SSAIL’s characteristically narrow process window as a reduction-re-oxidation competition rather than a mere damage threshold, predicts a metal-ion redox ladder (Ni^2+^/Cu^2+^/Ag^+^/Pd^2+^/Au^3+^) ordered by standard potential, and identifies electron paramagnetic resonance (EPR)-active spin defect densities as the quantity to correlate with plating yield [47]. The underlying activation-then-electroless-plating sequence is itself a standard route to selective metal deposition, applied for example to coat three-dimensional laser-fabricated structures with metal [48].

### 3.3. Reduced Titania with Gold as a Photoanode for the Oxygen Evolution Reaction

**Figure 3:**
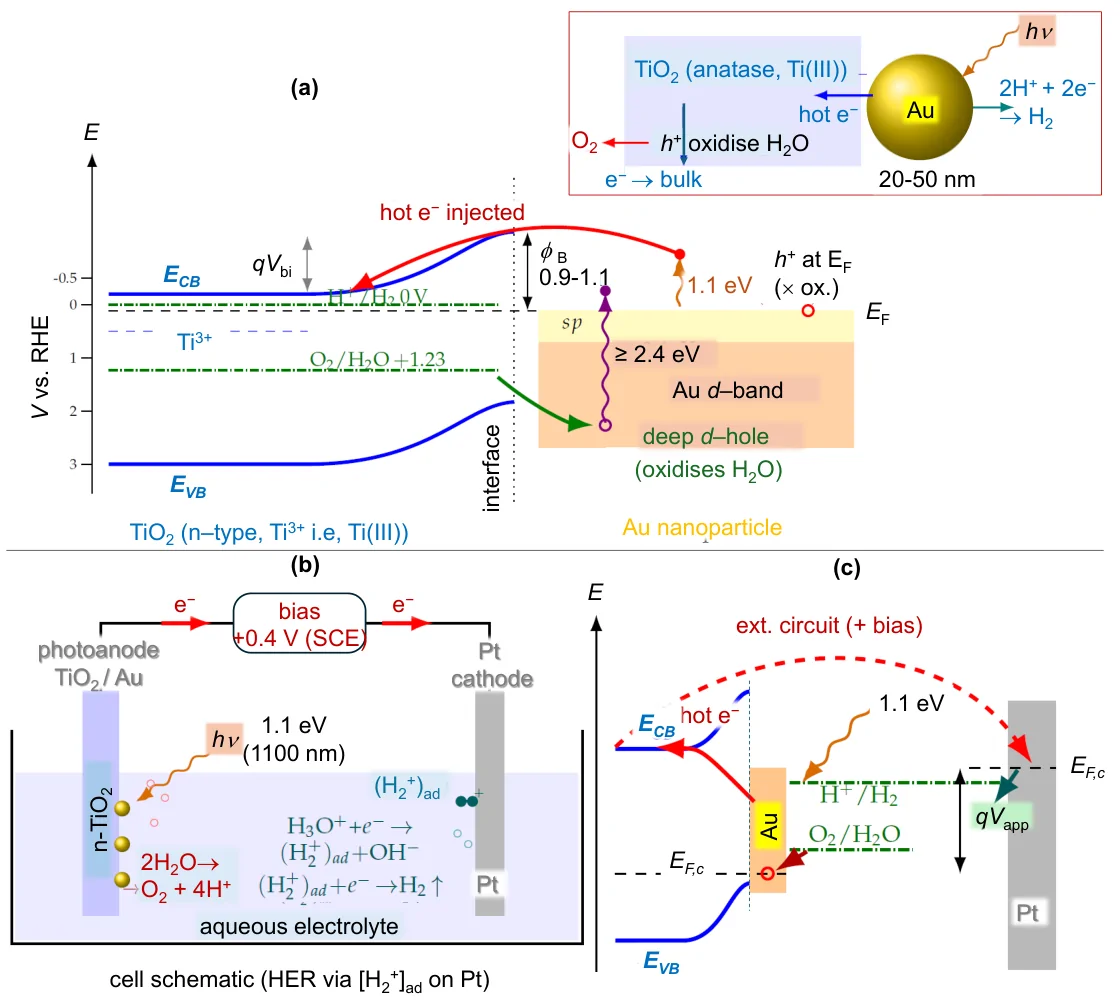
Au-decorated titania. (**a**) Real-space arrangement and reactions. (**b**) Interface energetics with the water redox levels on the same scale (vs. RHE; $H^{+}/H_{2}$ at 0 V just below $E_{CB}$; $O_{2}/H_{2}O$ at +1.23 V mid-gap). Two plasmonic channels are contrasted: an *IR intraband* excitation (∼1.1 eV) lifts an sp electron enough to clear $\phi_{B}$ and inject into the ${\mathrm{TiO}}_{2}$ conduction band, but the hole left near $E_{F}$ is *not* positive enough to oxidise water (×); only an *interband* d→sp excitation (≥2.4 eV) creates a deep *d*-band hole (∼+2.7 V vs. RHE) that can oxidise water (✓). (**c**) Biased cell: an anodic bias (+0.4 V vs. SCE) plus a separate Pt cathode let 1.1 eV (1100 nm) photons drive photocurrent. Injected electrons exit through the circuit to the Pt cathode, where $H_{2}$ evolution proceeds through the adsorbed molecular ion ${\left(H_{2}^{+}\right)}_{ad}$ ($H_{3}O^{+}$ + $e^{-}\;\to {\left(H_{2}^{+}\right)}_{ad}+$ ${\mathrm{OH}}^{-}$; ${\left(H_{2}^{+}\right)}_{ad}+e^{-}\;\to$$H_{2}$), while biased holes oxidise water at the ${\mathrm{TiO}}_{2}$/Au anode. The right diagram shows the two-electron cathodic ladder via ${\left(H_{2}^{+}\right)}_{ad}$ near 0 V (RHE).

The Ti(III)-rich titania becomes the n-type substrate of a plasmonic photoanode (Figure 3). Stoichiometric anatase is a semiconductor with an empty conduction band 3.2 eV up; the Ti^3+^/vacancy donors make it n-type and conductive, set up the depletion field/Schottky barrier, and add sub-band states that receive the injected electron, all prerequisites for a working electrode [20,43,44]. Gold (20–50 nm) adds a plasmon at ∼520–550 nm and a Schottky junction ($\phi_{B}\approx 0.9$–1.1 eV): sp hot electrons above $\phi_{B}$ are injected into the conduction band, while the Au/titania Schottky junction serves to separate these injected electrons from the *d*-band holes; hydrogen is evolved on a separate Pt cathode (Figure 3c), not on the gold [34,44]. A 1.1 eV (1100 nm) photon just lifts an sp electron over $\phi_{B}$, but the hole it leaves near $E_{F}$ cannot oxidise water. Only an interband d→sp excitation (≥2.4 eV) leaves a deep, oxidising *d*-band hole (Figure 3b): again the electron/hole (reduce/oxidise) partition.

**Plasmonic photocatalysis and the surface-normal field.** Decorating titania with plasmonic Au (or Ag) turns it into a visible-light photocatalyst, and action-spectrum analysis established that the photo-catalytic rate tracks the localized-plasmon absorption band of the metal—direct evidence that the chemistry is driven by plasmon-induced charge transfer into the oxide rather than by heating [33,42]. The part of the plasmonic near-field does the injecting is not arbitrary. For a metal nanoparticle sitting on a substrate, it is the depolarised field component normal to the interface (the $E_{z}$ component) that couples charge across the metal-semiconductor junction. For asymmetric spheroidal gold or silver nanoparticles on a dielectric substrate, we showed that the frequency- and polarisation-dependent optical response is governed by this perpendicular component, and that it is the surface-normal $E_{z}$ field, not the in-plane field, that is active in the measured action spectra [49]. Physically, $E_{z}$ is concentrated in the few-nanometer presurface region of the oxide where the band bending is strongest, so an oscillation perpendicular to the interface most efficiently drives hot electrons over the Schottky barrier into the conduction band [43].

A practical corollary, relevant both to the photoanode here and to the silicon detector below, is that the geometry should be chosen to maximise the surface-normal field: nanoparticles or nanorods with their long axis (or asymmetry) out of the surface plane, oblique or unpolarised illumination, and arrays engineered to concentrate $E_{z}$ at the contact all enhance the injecting channel, whereas purely in-plane (transverse) polarisation couples poorly to across-barrier transfer even when strongly absorbed. The match between the photo-response action spectrum and the plasmon resonance is therefore a signature that the normal-field charge-transfer channel, not mere plasmonic heating, dominates the activity.

Importantly, the practical potential of the OER is not the formal 1.23 V. Being a four-electron reaction, oxygen evolution on real surfaces cascades through intermediates, each with its own potential. All potentials below are reported on the reversible hydrogen electrode (RHE) scale, on which the proton-coupled couples are pH-invariant (Table 1). The two-electron peroxide stage (2 H_2_O → H_2_O_2_ + 2 H^+^ + 2 e^−^, E≈+1.78 V vs. RHE) sets the realistic onset [50,51]: H_2_O_2_ reappearing, now as the oxidation intermediate. Notably, the one-electron route is steeper still: formation of the HO^•^ radical requires a potential as high as +2.73 V vs. RHE [41]. The fewer electrons transferred per step, the higher the price per electron: 1 e^−^ (+2.73 V, HO^•^) > 2 e^−^ (+1.78 V, H_2_O_2_) > 4 e^−^ (+1.23 V, O_2_). On the energy ledger of Figure 3b, only the deep *d*-band holes (∼+2.7 V vs. RHE) are energetic enough to open the radical channel, whereas holes near $E_{F}$ of gold cannot drive even the four-electron reaction. The correction changes the absolute numbers but not the argument, which is built throughout on differences between potentials: these differences are pH-invariant, so the ladder (1 e^−^ HO^−^ > 2 e^−^ H_2_O_2_ > 4 e^−^ O_2_) and the 1.23 V thermodynamic voltage of water splitting hold at every pH. A single 1.1 eV photon therefore cannot split water unaided. In the biased cell of Nishijima et al. [44] (Figure 3c), an anodic bias (+0.4 V, SCE) and a Pt cathode supply the deficit. Electrons injected into the TiO_2_ conduction band are conducted through the external circuit to the Pt cathode, where they are taken up in the hydrogen evolution reaction (HER), while the loop is closed by ion transport in the electrolyte; the current is continuous around the circuit, so the anodic and cathodic rates are equal at all times. The OER proceeds at the TiO_2_/Au anode, and H_2_ evolves at Pt. Following Juodkazytė et al. [52], the cathodic step runs through the adsorbed dihydrogen cation, H_3_O^+^+ e^−^$\;\to {\left({H_{2}}^{+}\right)}_{ad}+$OH^−^ then $({H_{2}}^{+})ad$ + e^−^→H_2_, the H-H^+^ bond paid for by H^+^ hydration so that the discharge is depolarised to ∼0 V (RHE). This mechanism of water splitting is consistent with the simultaneously occurring mass and charge changes upon HER [52]. Fully plasmon-driven (unbiased) water splitting has been demonstrated when charge separation is engineered, e.g., in the autonomous device of Mubeen et al. [53].

### 3.4. Plasmonic Detection on Silicon Below the Band Gap

**Figure 4:**
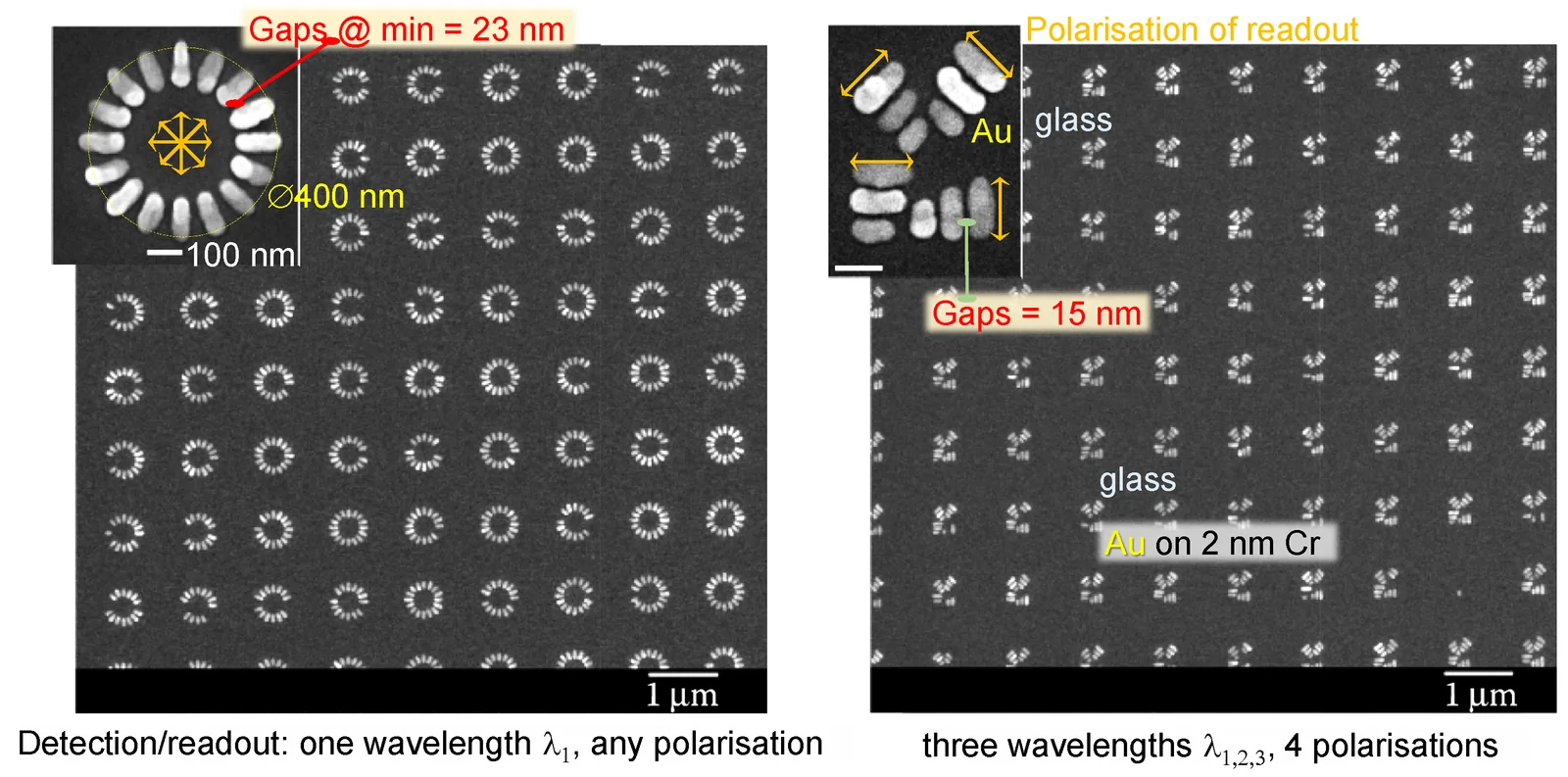
Electron-beam-lithography-defined structures for read-out of wavelength–polarisation by scattering/reflectance/transmittance. The thickness of Au was ∼40 nm, made by a lift-off process on glass. Note that the same structures can be made on Si substrates for detectors (discussed next, below). Such patterns were proposed as optical memory bits for multi-wavelength and polarisation multiplexing [43].

The same electron-beam lithography (EBL)-defined plasmonic antennas used to read out wavelength and polarisation on glass (Figure 4) can be placed on silicon to build detectors. Here the metal-semiconductor physics turns from chemistry to photocurrent.

**Figure 5:**
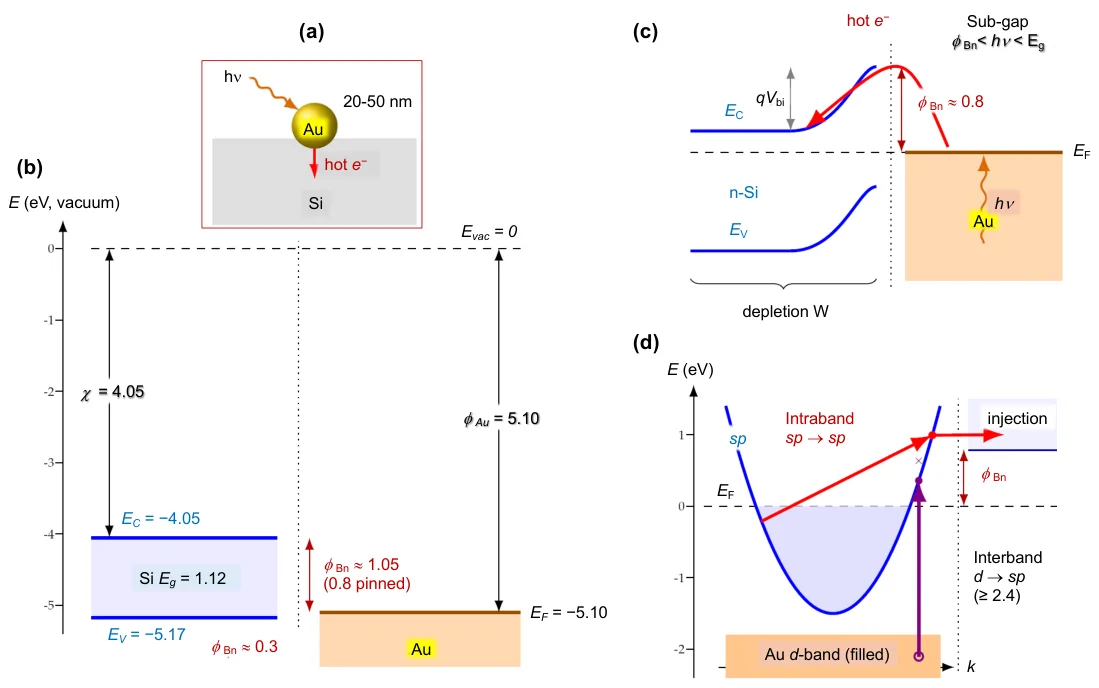
Au/Si hot carriers. (**a**) Real space: Au nanoparticle on Si. (**b**) Flat-band alignment (vacuum- referenced); the electron affinity of Si (χ=4.05 eV) and the work function of Au (ϕ=5.10 eV) are standard literature values [55,56]. (**c**) Equilibrium with upward band bending (depletion *W*, $qV_{bi}$); a sub-band-gap photon makes a hot electron in Au that is emitted over $\phi_{Bn}$ into Si. (**d**) Gold E(k): the sp band crosses $E_{F}$ and the filled *d*-band lies ∼2 eV below. An *intraband* (sp→sp, plasmon-assisted) excitation lifts an electron above $E_{F}$ and above $\phi_{Bn}$, so it injects into the Si conduction band (red); an *interband* (d→sp, ≥2.4 eV) excitation puts the electron only just above $E_{F}$ (below the barrier, ×) while leaving a deep *d*-band *hole* (violet). Hence the injected electrons are sp in character; the *d*-band supplies energetic holes, and in the sub-band-gap window only the sp channel operates.

The relevant levels (vacuum-referenced) are Si χ=4.05 eV, $E_{g}=1.12$ eV ($E_{C}=-4.05$, $E_{V}=-5.17$ eV) and Au ϕ=5.10 eV ($E_{F}=-5.10$ eV), giving an electron Schottky barrier $\phi_{Bn}=\phi_{Au}-\chi_{Si}\approx 1.05$ eV (Schottky-Mott; ∼0.8 eV with Fermi-level pinning) and a small hole barrier $\phi_{Bp}\approx 0.3$ eV (Figure 5). After contact, the Fermi levels equilibrate and the n-Si bands bend over a depletion width *W*, leaving $\phi_{Bn}$ unchanged. Plasmon decay in 20–50 nm Au gives hot electrons up to ∼ℏω above $E_{F}$; those exceeding $\phi_{Bn}$ are emitted over the barrier into Si and collected by the built-in field. Crucially, this works for $\phi_{Bn}<h\nu <E_{g}$ (∼0.8–1.12 eV, λ≈1.1–1.5μm), i.e., *below* the silicon gap, internal photoemission, the basis of Au/Si Schottky hot-electron detectors for the near-infrared [35,36,54]. The injected carriers are *sp***-band** electrons: the filled *d*-band (∼1.8–2.4 eV below $E_{F}$) supplies energetic electrons only via interband (d→sp, ≥2.4 eV) excitation, which places the electron just above $E_{F}$ (below the barrier) while leaving a deep *d*-band *hole* (Figure 5d). In the sub-band-gap window, only the sp channel operates, the mirror image of the titania case, where the *d*-band holes did the oxidation chemistry.

**Polarisation and wavelength sensitivity.** Because a nanorod’s longitudinal plasmon scales with its aspect ratio, an array of rods is intrinsically wavelength- and polarisation-selective, and this selectivity is carried directly into the photocurrent. In the titania photoanode of Figure 2 the Au-nanorod pattern shows distinct transverse and longitudinal extinction bands (here near 680 and 1000 nm) whose relative weight follows the incident polarisation; the plasmon-assisted photocurrent measured by Nishijima et al. accordingly peaks at the wavelength and polarisation that maximise the longitudinal resonance, so the same electrode effectively reports both the colour and the polarisation of the sub-band-gap light it converts [43,44]. Transplanting the identical lithographic patterns onto silicon turns this into a hot-electron photodetector with built-in spectral and polarisation discrimination: rods of different length resonate and therefore inject over the Schottky barrier at different wavelengths, while rods of a given length respond only to light polarised along their long axis, the orientation that also maximises the surface-normal injecting field discussed above for the photoanode. An array interleaving several rod lengths and orientations thus provides a set of photocurrent channels, each tuned to a particular wavelength-polarisation combination, realising on a single chip the filterless wavelength-polarisation read-out sketched in Figure 4. The price is the familiar one of internal photoemission, a modest quantum efficiency traded for operation below the silicon band gap and for intrinsic spectral and polarisation selectivity.

## 4. Conclusions

The thread running through these three settings is that light simultaneously drives reduction and oxidation. In femtosecond processing of an aqueous anatase suspension the breakdown plasma reduces the surface to Ti^3+^/TiO_2−x_ while the radiolytic ROS (HO^•^, H_2_O_2_) re-oxidise it; intensity clamping (∼$10^{13}$ W cm^−2^) together with this redox competition caps the reduction at “blue,” leaving the bulk anatase intact and the Ti^3+^ confined to the surface. Decorating the reduced oxide with gold tunes its visible–near-IR absorption and, through the surface-normal ($E_{z}$) plasmonic field, injects carriers across the interface, so that the photoactivity exceeds what reduction alone provides. The same Ti^3+^ donors make the oxide a conductive n-type photoanode on which sp hot electrons drive reduction and *d*-band holes drive the OER.

A small applied bias completes the cell: current is continuous around the closed circuit, and hydrogen evolves at the platinum cathode through the adsorbed (H_2_^+^)_ad_ intermediate. Finally, the identical Au/semiconductor physics transferred to silicon yields a hot-electron photo-detector that responds below the silicon band gap (∼1.1–1.5μm): sp electrons are emitted over a ∼0.8 eV Schottky barrier while the *d*-band again supplies the oxidising holes, and patterning the gold as nanorods of chosen length and orientation renders the photocurrent wavelength- and polarisation-selective without filters. In every case it is the partition of the absorbed plasmonic energy into sp electrons and *d* holes (into reductants and oxidants) that determines what the device can do.

Several challenges remain open. The long-term stability of the blue TiO_2−x_ under aerated conditions and the durability of Au nanoparticles during extended photocatalytic operation are yet to be established at device-relevant scales. Looking ahead, suppressing the competing oxidative channel during fs-laser synthesis could push the reduction deeper toward black TiO_2−x_, while integrating the Au-nanorod geometry directly onto Si substrates offers a route to filterless near-infrared imaging arrays.

## Figures and Tables

**Table 1:** Standard reduction potentials of the couples discussed, on the standard hydrogen electrode (SHE) scale at pH 0 and pH 7 and on the pH-invariant reversible hydrogen electrode (RHE) scale. Proton-coupled couples shift by −59 mV per pH unit on SHE and cancel on RHE; Ag^+^/Ag^0^ transfers no proton and is the one couple that must remain on SHE.

Couple	Half-Reaction (Reduction)	*E* vs. SHE (pH 0)	*E* vs. SHE (pH 7)	*E* vs. RHE
H^+^/H_2_	2 H^+^ + 2 e^−^ → H_2_	0.000	−0.414	0.000
O_2_/H_2_O (4 e^−^)	O_2_ + 4 H^+^ + 4 e^−^ → 2 H_2_O	+1.229	+0.815	+1.229
H_2_O_2_/H_2_O (2 e^−^)	H_2_O_2_ + 2 H^+^ + 2 e^−^ → 2 H_2_O	+1.776	+1.362	+1.776
HO^•^/H_2_O (1 e^−^)	HO^•^ + H^+^ + e^−^ → H_2_O	+2.730	+2.316	+2.730
Ti(IV)/Ti(III), oxide	2 TiO_2_ + 2 H^+^ + 2 e^−^ → Ti_2_O_3_ + H_2_O	−0.091	−0.505	−0.091
Ag^+^/Ag^0^	Ag^+^ + e^−^ → Ag^0^	+0.799	+0.799	+1.21

## Data Availability

All data are presented in this perspective paper.
